# Masson’s tumor involving the hand: A case report

**DOI:** 10.1016/j.ijscr.2020.04.069

**Published:** 2020-05-08

**Authors:** Mohammed A. Almarghoub, Qutaiba N.M. Shah Mardan, Ahmed S. Alotaibi, Norhan K. Ahmed, Moraya S. Alqahtani

**Affiliations:** a King Faisal Specialist Hospital and Research Center, Department of Surgery, Plastic and Reconstructive Surgery Section, Riyadh, Saudi Arabia; b Alfaisal University, College of Medicine, Riyadh, Saudi Arabia

**Keywords:** Masson’s tumor, Intravascular papillary endothelial hyperplasia, IPEH, Hand, Case report

## Abstract

**Introduction:**

Masson’s tumor or IPEH represents a rare exuberant endothelial proliferation within a thrombus through an uncomprehended phenomenon. Being reported for the 1st time in Saudi Arabia, plastic surgeons should keep it in the list of differential diagnosis.

**Case:**

We report a case of 17-year-old-girl who presented with a 6-month-old, painful mass on the volar side of her left 4th MCP. Radiology was inconclusive. Histopathology reported Masson’s tumor following surgical excision with good functional outcome and no recurrence.

**Discussion:**

Comprising 2%–4% of overall skin vascular tumor and with no identifying clinical or radiological feature, IPEH poses as a diagnostic challenge. The literature reports similar tumors in the hand with different locations and presentations. Surgical excision remains the cornerstone of management, yet the role of radiotherapy remains undefined. Incomplete excision may result in recurrence, which requires a consensus on the extend of marginal excision. Rare cases of recurrence were reported. Histopathology is the only reliable method of diagnosis.

**Conclusion:**

We present the 1st reported case of IPEH involving the hand in Saudi Arabia. Plastic surgeons should be aware of such rare conditions and be able to differentiate them from relatively identical yet more sinister tumors, especially angiosarcoma.

## Highlights

• Masson’s tumor or IPEH is a rare benign tumor composed of reactive endothelial cells in a thrombus.
• Angiosarcoma may mimick Masson’s tumor, thus ruling it out is imperative.
• Surgical resection conveys excellent outcome and prognosis, with handful of recurrence cases.

## 1 Introduction

Masson’s tumor, otherwise known as intravascular papillary endothelial hyperplasia (IPEH), is a rare, benign vascular tumor originating from the reactive proliferation of injured endothelial cells within a thrombus. Despite showing a clear predilection for the head, neck, and extremities, Masson’s tumor has been recently reported in few other peculiar locations including the eyelid, parotid gland, sinonasal cavity, and kidneys, with no pathognomonic features [1]. Given that this is the 1st report of such a condition in Saudi Arabia and the necessity for the plastic surgeon awareness of this commonly misdiagnosed lesion, we present a case of a Masson’s tumor arising in the finger of a 17-year-old-female, following the SCARE grid [2].

## 2 Case

**Fig. 1:**
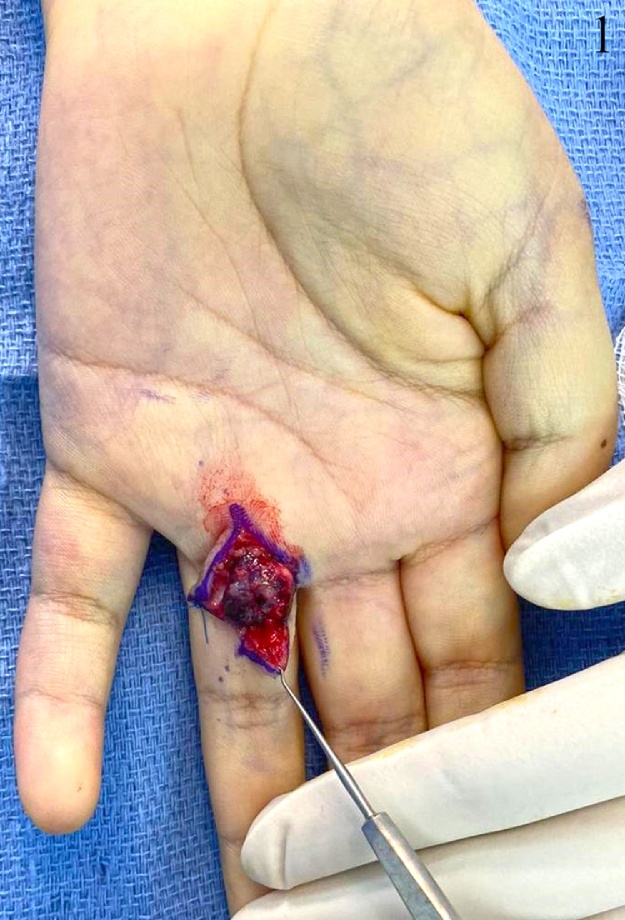
This is an intraoperative image of the lesion.

**Fig. 2:**
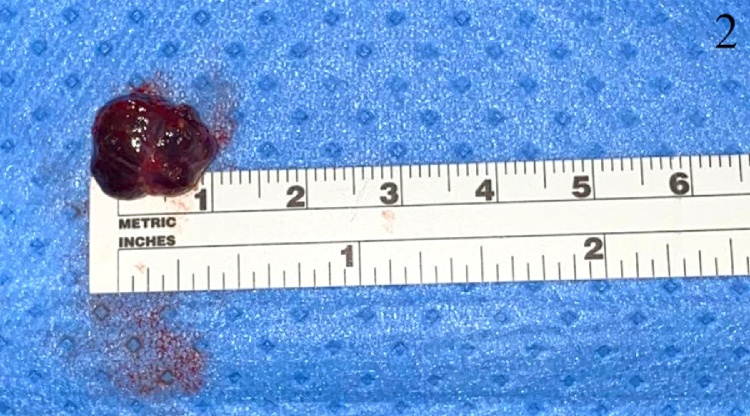
The lesion following excision.

**Fig. 3:**
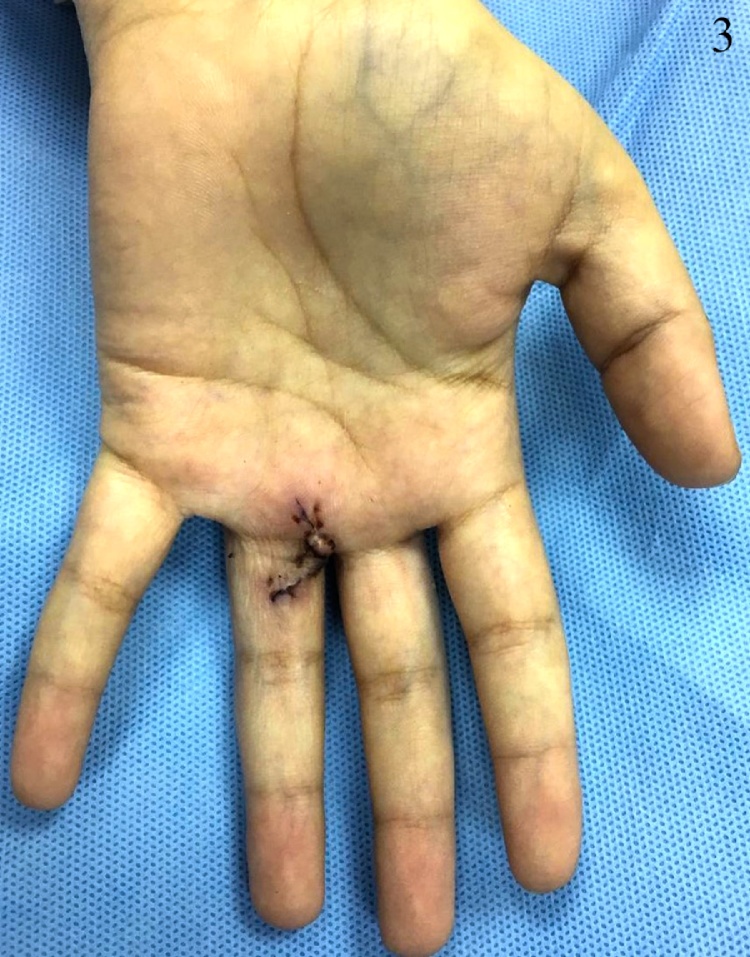
The wound approximately two weeks following excision.

A 17-year-old lady, not known to have any medical illnesses, presented to the plastic surgery clinic in our institute with a painful, progressively growing mass on the left middle finger. Six months prior to presentation, it has started as a painless, green mass, after which she was referred to us. Physical examination revealed a dark red swelling on the palmar side of the left 4th MCP with preserved range of motion and sensation. Capillary refill or sensation were not compromised. Magnetic resonance imaging (MRI) with gadolinium showed a subcutaneous rounded soft tissue lesion with internal high T2 and T1 signal intensity. Local excision was performed under general anesthesia. Grossly measuring 1 × 1 × 0.5 cm, the lesion was well-circumscribed, brown-black in color, and rubbery in consistency. Pathology report indicated an organizing thrombus with papillary endothelial hyperplasia or Masson’s tumor. With no peri-operative or post-operative complications, no recurrence was noticed 1 year later during a follow-up visit (Fig. 1, Fig. 2, Fig. 3).

## 3 Discussion

First documented in 1923, Pierre Masson, a French pathologist, described a lesion in the lumen of an ulcerated hemorrhoidal vein and labeled it "hémangio-endothéliome végétant intravasculaire”, or better known today as Masson’s tumor or IPEH [3]. Comprising 2%–4% of all benign and malignant vascular tumors of the skin and subcutaneous tissues, this lesion had demonstrated slight inclination towards women; a ratio of 1.2:1 compared to men [4,5]. It is further subdivided into three categories based on the origin. Type I represents a de-novo incident stemming within normal blood vessels, while type II can develop from a pre-existing vascular process: hemangioma, pyogenic granuloma, or a hematoma. Type III, the least common variant, has an extravascular location and generally arises from post-traumatic hematomas [6]. The pathogenesis of the lesion remains uncovered. It is postulated that Masson’s tumor growth is driven by endothelial basic fibroblast growth factor, released by macrophages [1]. Hormonal involvement might be implicated, which may explain the tendency to develop in women [7]. Although many cases present following a trauma, only 4% of the patients report such history [8], as the scenario in our case where the patient denied any history of trauma.

The diagnosis of Masson’s tumor is challenging owing to its clinical and radiological resemblance to other vascular tumors, especially angiosarcoma (See Table 1). Masson’s tumor may appear clinically as a sharply demarcated, firm or tender mass with slight elevation and slowly progressive growth. Discoloration of the overlying skin or mucous membranes could take different colors including blue and red [1]. It can be painless or painful [9], with the latter being the situation in our case. Other than the skin and subcutaneous tissue of the hands, Masson’s tumor may arise adherent to a common digital nerve, originating from its perineural vasculature [9]. It may mimic a neurogenic tumor with positive Tinel signs and paresthesia across the distribution of the affected neighboring digital nerve [10].

**Table 1:** This table compares different features of Masson’s tumor with angiosarcoma.

	Masson’s tumor (IPEH)	Angiosarcoma
Nature	Rare benign vascular tumor of soft tissues [1]	Aggressive, malignant subtype of soft-tissue sarcomas [17]
Location	Head, neck and, extremities [1]	Skin, scalp, breast, liver, spleen, and deep tissues [17]
Causes	De novo, vascular injury, and post-traumatic hematomas [6]	Lymphedema, radiation exposure and exposure to polyvinyl chloride, arsenic, and thorium dioxide [17]
Clinical Presentation	Well-defined superficial papules or deep nodules. Demonstrates progressive growth with discoloration of overlying skin [1]	Bruise-like patches, violaceous nodules or plaques or enlarged painful mass [18]
MRI	minimally heterogeneous mass on T1, mostly isointense to muscle. T2-weighted images show a centrally heterogeneous iso-to-slightly high signal intensity mass, completely or incompletely surrounded by peripheral high signal intensity areas [12]	Intermediate T1 signal intensity, with possible areas of hyperintensity indicating hemorrhage, and high T2 signal intensity. Enhances with intravenous contrast [17]
Management	Local excision (curative) [1] Undefined role for radiotherapy [16]	Surgical, chemotherapeutic, and radiotherapy [17]
Histopathology	Hyperplastic endothelial cells forming a papillary growth completely confined within the vascular lumen [1]	Histopathological variants include infiltration into subcutis, papillary endothelial hyperplasia, prominent nucleoli, mitotic figures, significant cytological atypia, and dissection of dermal collagen [19]
Immunohistochemistry	CD31, CD34, SMA and factor VIII-related antigen are indicative of IPEH [1]	CD105 [1]
Metastatic potential	None [1]	Nodal and distant metastases are present and confer poor prognosis [17]
Recurrence	Rare [16]	Locoregional recurrence is frequent [17]

Ultrasound can detect one or more vessels associated with the lesion, differentiating IPEHs from other soft tissue masses [11]. MRI demonstrates a minimally heterogeneous mass on T1, mostly isointense to muscle, while T2-weighted images show a centrally heterogeneous iso-to-slightly high signal intensity mass, completely or incompletely surrounded by peripheral high signal intensity areas. post contrast T1-weighted images show heterogeneous enhancement. However, the number of patients in the study is quite minimal and represent solely type I. Moreover, there is inconsistency of findings in MRI among other studies [12]. Henceforth the pivotal role of histopathology in diagnosis. Angiosarcoma arising within vascular lumen is an extremely uncustomary finding, a key histopathological difference from Masson’s tumor, with the exclusion of type III which tends to be extravascular [7].

Treatment is mainly through surgical resection, which can be curative and result in excellent outcome and prognosis with slim chances of recurrence, especially in type II. As of yet, there is no consensus regarding the margins of resection as generally there is no indication for wide margins of excision; however, incomplete resection may result in recurrence [13]. Occasions of recurrence had been rarely reported in the literature [14,15] and are intimately associated with incomplete excision or an underlying co-existing vascular tumor [16]. Radiotherapy was employed in the management of this tumor, nonetheless defined indications are lacking. Anthony et al. used radiotherapy in clearing a partially excised, recurrent IPEH encasing the ulnar neurovascular bundle in an ulnar-artery-predominant hand. This decision was on the premise of preserving the ulnar neurovascular bundle and resulted in excellent outcome with no recurrence [16].

## 4 Conclusion

Masson’s tumor or (IPEH), is being encountered by the plastic surgeons more and more, albeit rarity. We narrate a case of a 17-year-old medically free girl with a mass in the hand, which was found to be Masson’s tumor after resection and histopathology analysis. No cardinal features can be pin-pointed at, in both clinical examination or radiology, that lead to IPEH. Consequently, it may not take a place in the differential diagnosis list of the treating surgeon.

## Declaration of Competing Interest

None.

## Sources of funding

None.

## Ethical approval

Exempted from the IRB approval.

## Consent

Written informed consent was obtained from the patient for publication of this case report and accompanying images. A copy of the written consent is available for review by the Editor-in-Chief of this journal on request.

## Author contribution

1. Mohammed Almarghoub: Manuscript writing and editing.
2. Qutaiba Shah Mardan: Manuscript writing and editing.
3. Ahmed Alotaibi: Manuscript writing and editing.
4. Norhan Ahmed: Manuscript writing and editing.
5. Moraya Alqahtani; Supervision; Treatment of the patient and manuscript editing.

## Registration of research studies

This paper does not require registry as it is a case report about a condition that has been discussed in prior papers. There is no additional harm to the patient nor an innovative intervention is being applied on the patient.

## Guarantor

Mohammed Almarghoub.

Qutaiba Shah Mardan.

## Provenance and peer review

Not commissioned, externally peer-reviewed.
